# Crystal structure and Hirshfeld surface analysis of 4-(2,6-di­chloro­benz­yl)-6-phenyl­pyridazin-3(2*H*)-one

**DOI:** 10.1107/S2056989019005139

**Published:** 2019-04-18

**Authors:** Fouad El Kali, Sevgi Kansiz, Said Daoui, Rafik Saddik, Necmi Dege, Khalid Karrouchi, Noureddine Benchat

**Affiliations:** a Laboratory of Applied Chemistry and Environment (LCAE), Department of Chemistry, Faculty of Sciences, University Mohamed Premier, Oujda 60000, Morocco; b Ondokuz Mayıs University, Faculty of Arts and Sciences, Department of Physics, 55139, Kurupelit, Samsun, Turkey; c Laboratory of Organic Synthesis, Extraction and Development, Faculty of, Sciences, Hassan II University, Casablanca, Morocco; d Laboratory of Plant Chemistry, Organic and Bioorganic Synthesis, URAC23, Faculty of Science, BP 1014, GEOPAC Research Center, Mohammed V University, Rabat, Morocco

**Keywords:** crystal structure, pyridazin, hydrogen bonding, π–π inter­actions, Hirshfeld surface analysis

## Abstract

The asymmetric unit of the title compound, C_17_H_12_Cl_2_N_2_O, contains one independent mol­ecule. The mol­ecule is not planar, the phenyl and pyridazine rings are twisted with respect to each other, making a dihedral angle of 29.96 (2)° and the di­chloro­phenyl ring is nearly perpendicular to the pyridazine ring, with a dihedral angle of 82.38 (11)°. In the crystal, pairs of N—H⋯O hydrogen bonds link the mol­ecules to form inversion dimers with an *R*_2_^2^(8) ring motif. The dimers are linked by C—H⋯O inter­actions, forming layers parallel to the *bc* plane. The inter­molecular inter­actions were investigated using Hirshfeld surface analysis and two-dimensional fingerprint plots, and the mol­ecular electrostatic potential surface was also analysed. The Hirshfeld surface analysis of the title compound suggests that the most significant contributions to the crystal packing are by H⋯H (31.4%), Cl⋯H/H⋯Cl (19.9%) and C⋯H/H⋯C (19%) contacts.

## Abstract 2

In the crystal, the mol­ecules are linked by a pair of N—H⋯O hydrogen bonds, forming inversion dimers with an 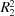
(8) ring motif. The dimers are linked by C—H⋯O hydrogen bonds, forming layers parallel to the *bc* plane and by weak π–π inter­actions, forming layers parallel to the *ab* plane.

## Chemical context   

Pyridazinone derivatives are biologically active heterocyclic compounds (Akhtar *et al.*, 2016 ▸). Diverse pyridazinone derivatives have been reported to possess a variety of biological activities (Thakur *et al.* 2010 ▸; Asif *et al.* 2015 ▸) such as anti­microbial (Sönmez *et al.* 2006 ▸), anti-inflammatory (Abouzid *et al.* 2008 ▸), analgesic (Gökçe *et al.* 2009 ▸), anti-HIV (Livermore *et al.* 1993 ▸), anti­hypertensive (Siddiqui *et al.* 2011 ▸), anti­convulsant (Sharma *et al.* 2014 ▸), cardiotonic (Wang *et al.* 2008 ▸), anti­histaminic (Tao *et al.* 2012 ▸), anti­depressant (Boukharsa *et al.* 2016 ▸), glucan synthase inhibitors (Zhou *et al.* 2011 ▸), phospho­diesterase (PDE) inhibitors (Ochiai *et al.* 2012 ▸) and herbicidal activity (Asif *et al.* 2013 ▸). We report herein the synthesis and the crystal and mol­ecular structures of the title compound, as well as an analysis of its Hirshfeld surfaces.
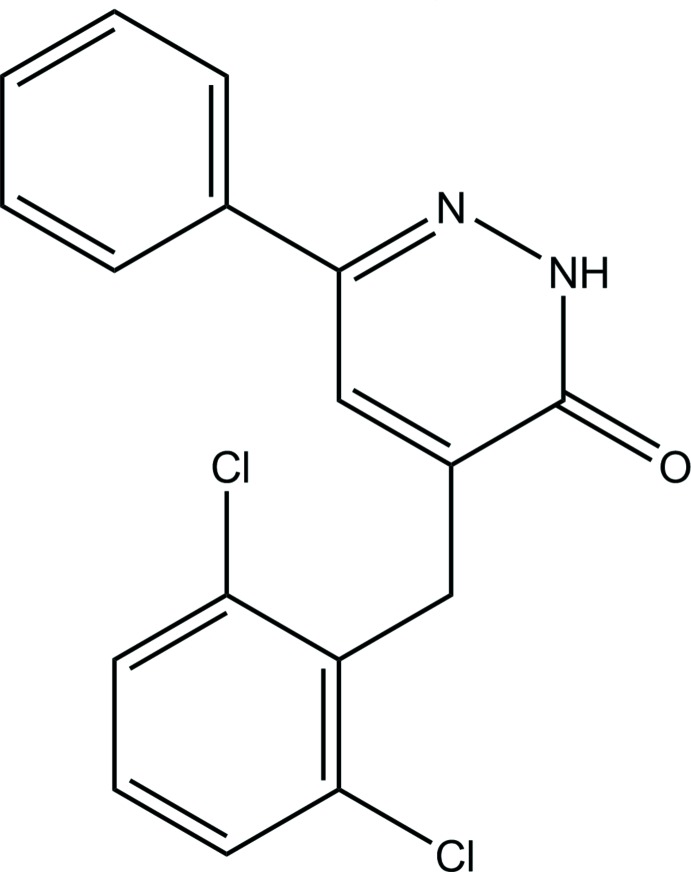


## Structural commentary   

**Figure 1:**
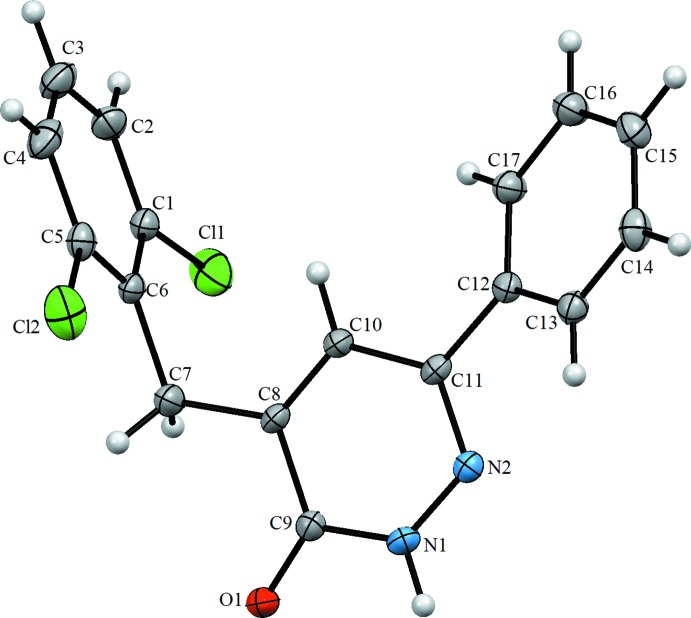
The mol­ecular structure of the title compound, with the atom labelling. Displacement ellipsoids are drawn at the 20% probability level.

As the mol­ecular structure of the title compound is illustrated in Fig. 1 ▸; the asymmetric unit contains one independent mol­ecule. The mol­ecule is not planar, the benzene ring (C12–C17) and the pyridazine ring are twisted relative to each other, making a dihedral angle of 29.96 (2)° and the phenyl ring (C1–C6) is nearly perpendicular to the pyridazine ring with a dihedral angle of 82.38 (11)° (Fig. 1 ▸). The C9=O1 bond length is 1.248 (4) Å while the C9—N1 and C11—N2 bond lengths are 1.360 (4) and 1.307 (4) Å, respectively.

## Supra­molecular features   

**Figure 2:**
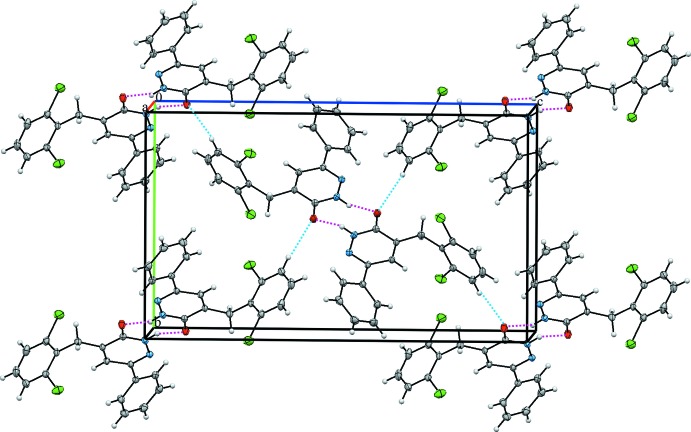
A view along the *a* axis of the crystal packing of the title compound. Dashed lines denote the N—H⋯O hydrogen bonds (Table 1 ▸) forming an inversion dimer with an 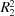
(8) ring motif. The C—H⋯O inter­actions are shown as blue dashed lines.

**Figure 3:**
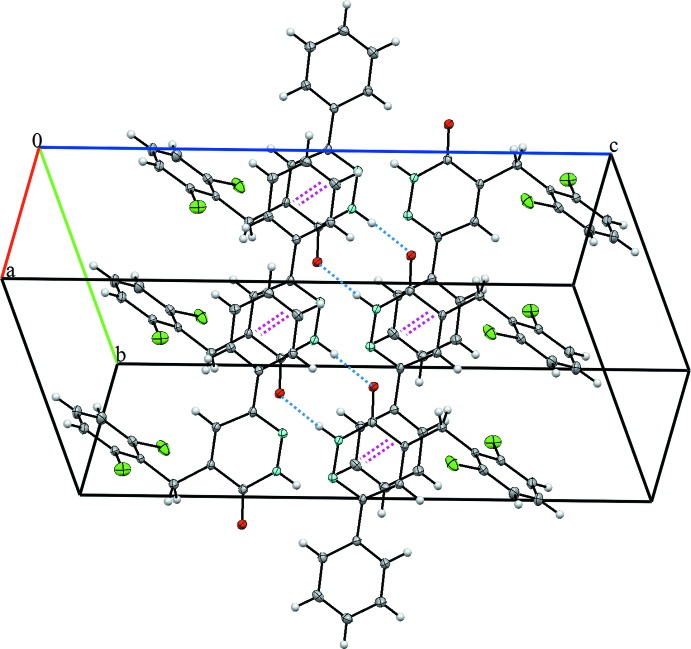
A view along the *a* axis of the crystal packing of the title compound. The hydrogen bonds (Table 1 ▸) are shown as dashed lines and the π–π inter­actions as pink dashed lines.

In the crystal, the mol­ecules are linked by a pair of N—H⋯O hydrogen bonds, forming inversion dimers with an 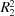
(8) ring motif (Table 1 ▸ and Fig. 2 ▸). The dimers are linked by C—H⋯O hydrogen bonds, forming layers parallel to the *bc* plane (Fig. 2 ▸) and by weak π–π [*Cg*1⋯*Cg*3 = 3.839 (2) Å; *Cg*1 and *Cg*3 are the centroids of the N1–N2/C9-C11 and C12–C17 rings, respectively] inter­actions, forming a three-dimensional structure (Fig. 3 ▸).

## Database survey   

**Figure 4:**
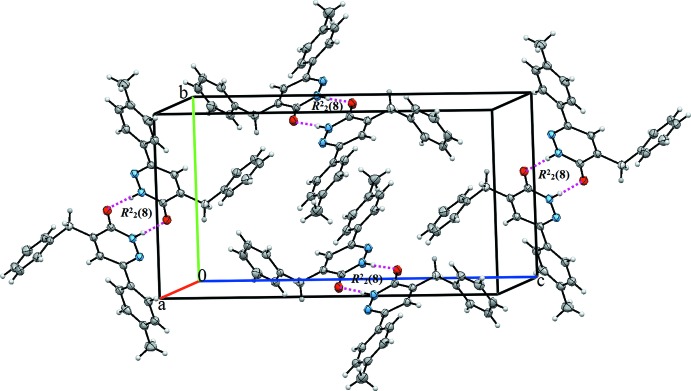
The crystal packing of YOTVIN (Oubair *et al.*, 2009 ▸). The N—H⋯O hydrogen bonds with an 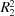
(8) graph set motif are shown as pink dashed lines.

**Figure 5:**
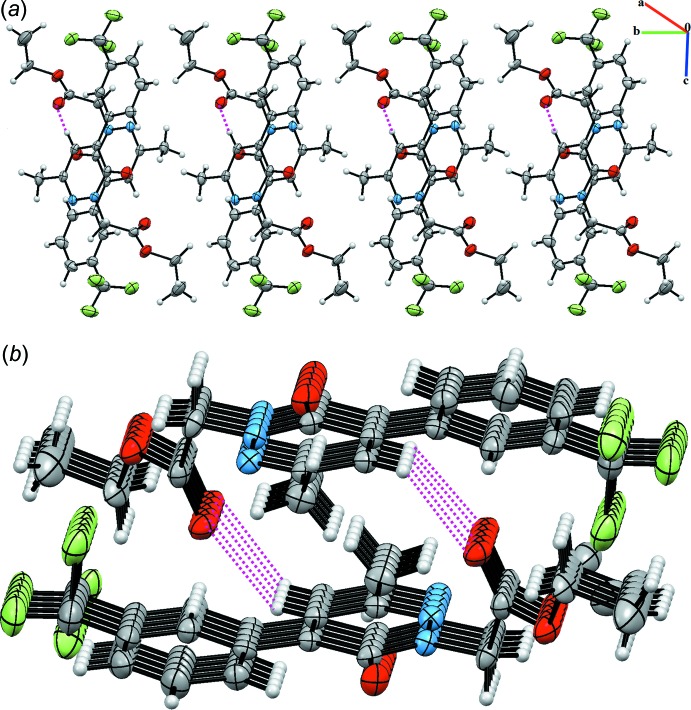
(*a*) A view of the dimers linked by C—H⋯O inter­actions forming layers parallel to the *bc* plane. (*b*) A view along the *c* axis of the crystal packing of QANVOR (Xu *et al.*, 2005 ▸). Dashed lines denote the inter­molecular C—H⋯O hydrogen bonds forming centrosymmetric dimers.

A search of the Cambridge Structural Database (CSD, version 5.40, update November 2018; Groom *et al.*, 2016 ▸) for the 4-phenyl­pyridazin-3(2*H*)-one skeleton yielded two hits: 4-benzyl-6-p-tolyl­pyridazin-3(2*H*)-one (YOTVIN; Oubair *et al.*, 2009 ▸) and ethyl 3-methyl-6-oxo-5-(3-(tri­fluoro­meth­yl)phen­yl)-1,6-di­hydro-1-pyridazine­acetate (QANVOR; Xu *et al.*, 2005 ▸). In YOTVIN, the mol­ecules are connected two by two through N—H⋯O hydrogen bonds with an 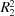
(8) graph-set motif, building a pseudo dimer arranged about the inversion center (Fig. 4 ▸). Weak C—H⋯O hydrogen bonds and weak offset π–π stacking inter­actions stabilize the packing. In QANVOR, the phenyl and pyridazinone rings are approximately coplanar with a dihedral angle of 4.84 (13)° and in the crystal, centrosymmetrically related mol­ecules form dimers through non-classical inter­molecular C—H⋯O hydrogen bonds (Fig. 5 ▸).

## Hirshfeld surface analysis   

**Figure 6:**
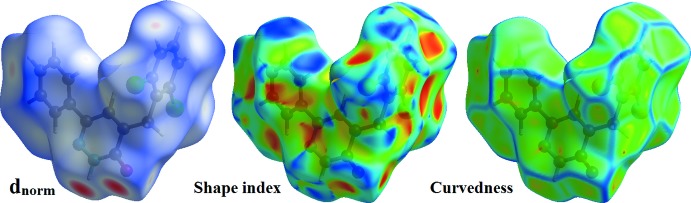
The Hirshfeld surfaces of the title compound mapped over *d*_norm_, shape-index and curvedness.

**Figure 7:**
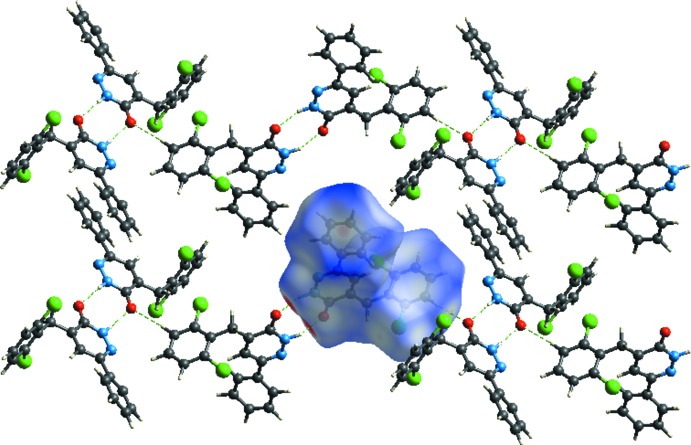
*d_norm_* mapped on Hirshfeld surfaces for visualizing the inter­molecular inter­actions of the title compound.

The Hirshfeld surface analysis (Spackman & Jayatilaka, 2009 ▸) and the associated two-dimensional fingerprint plots (McKinnon *et al.*, 2007 ▸) were performed with *CrystalExplorer17* (Turner *et al.*, 2017 ▸). In Fig. 6 ▸, the mappings of *d_norm_*, shape-index and curvedness for the title compound are shown. Fig. 7 ▸ illustrates the Hirshfeld surface of the mol­ecule in the crystal, with the evident hydrogen-bonding inter­actions indicated by intense red spots.

**Figure 8:**
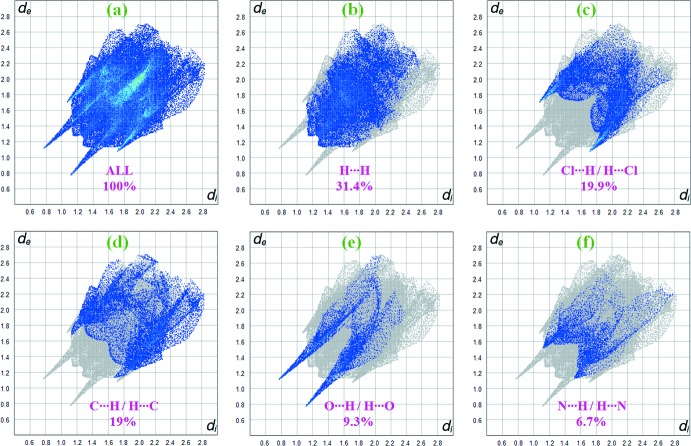
Two-dimensional fingerprint plots for the title compound, with a *d_norm_* view and the relative contribution of the atom pairs to the Hirshfeld surface.

Fig. 8 ▸ *a* shows the two-dimensional fingerprint of the sum of the contacts contributing to the Hirshfeld surface represented in normal mode. Two-dimensional fingerprint plots provide information about the major and minor percentage contributions of inter­atomic contacts in the compound. The blue colour refers to the frequency of occurrence of the (*d*_i_, *d*_e_) pair and the grey colour is the outline of the full fingerprint. The fingerprint plot in Fig. 8 ▸ *b* shows that the H⋯H contacts clearly make the most significant contribution to the Hirshfeld surface (31.4%). In addition, Cl⋯H/H⋯Cl, C⋯H/H⋯C, O⋯H/H⋯O and N⋯H/H⋯N contacts contribute 19.9%, 19%, 9.3% and 6.7%, respectively, to the Hirshfeld surface. In particular, the O⋯H/H⋯O contacts indicate the presence of inter­molecular N—H⋯O and C—H⋯O inter­actions. Much weaker Cl⋯C/C⋯Cl (6.1%) and C⋯C (3.7%) contacts also occur.

**Figure 9:**
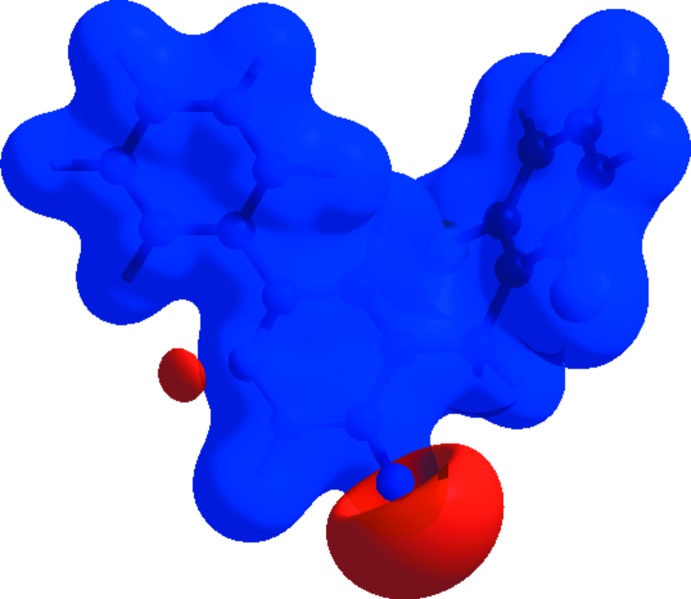
A view of the mol­ecular electrostatic potential for the title compound in the range −0.0500 to 0.0500 a.u. using the 6–31 G(d,p) basis set by the DFT method.

A view of the mol­ecular electrostatic potential, in the range −0.0500 to 0.0500 a.u. using the 6-31G(d,p) basis set with DFT method, for the title compound is shown in Fig. 9 ▸, where the N—H⋯O hydrogen-bond donors and acceptors are shown as blue and red areas around the atoms related with positive (hydrogen-bond donors) and negative (hydrogen-bond acceptors) electrostatic potentials, respectively.

## Synthesis and crystallization   

To a solution (0.15 g, 1 mmol) of 6-phenyl-4,5-di­hydro­pyridazin-3(2*H*)-one and (0.18 g, 1 mmol) of 2,6-di­chloro­benzaldehyde in 30 ml of ethanol, sodium hydroxide 10% (0.5 g, 3.5 mmol) was added. The solvent evaporated under vacuum, the residue was purified through silica gel column chromatography using hexa­ne/ethyl acetate (7:3 *v*/*v*). Single crystals were obtained by slow evaporation at room temperature.

## Refinement   

Crystal data, data collection and structure refinement details are summarized in Table 2 ▸. The nitro­gen-bound H atom was located in a difference-Fourier map and refined subject to a DFIX restraint of N—H = 0.86 Å. The C-bound H atoms were positioned geometrically and refined using a riding model: C—H = 0.93–0.97 Å with *U*_iso_(H) = 1.2*U*_eq_(C).

## Supplementary Material

Crystal structure: contains datablock(s) I. DOI: 10.1107/S2056989019005139/dx2016sup1.cif

Structure factors: contains datablock(s) I. DOI: 10.1107/S2056989019005139/dx2016Isup2.hkl

Supporting information file. DOI: 10.1107/S2056989019005139/dx2016Isup3.cml

CCDC reference: 1896404

Additional supporting information:  crystallographic information; 3D view; checkCIF report

## Figures and Tables

**Table 1:** Hydrogen-bond geometry (Å, °)

*D*—H⋯*A*	*D*—H	H⋯*A*	*D*⋯*A*	*D*—H⋯*A*
N1—H1⋯O1^i^	0.86	2.04	2.839 (4)	155
C2—H2⋯O1^ii^	0.93	2.66	3.581 (6)	172

Symmetry codes: (i) 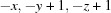; (ii) 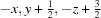.

**Table 2:** Experimental details

Crystal data	
Chemical formula	C_17_H_12_Cl_2_N_2_O
*M*_r_	331.19
Crystal system, space group	Monoclinic, *P*2_1_/*c*
Temperature (K)	296
*a*, *b*, *c* (Å)	5.8511 (6), 12.5544 (15), 21.069 (2)
β (°)	92.666 (8)
*V* (Å^3^)	1546.0 (3)
*Z*	4
Radiation type	Mo *K*α
μ (mm^−1^)	0.42
Crystal size (mm)	0.74 × 0.29 × 0.05

Data collection	
Diffractometer	Stoe *IPDS* 2
Absorption correction	Integration (*X-RED32*; Stoe & Cie, 2002 ▸)
*T*_min_, *T*_max_	0.844, 0.973
No. of measured, independent and observed [*I* > 2σ(*I*)] reflections	8675, 2726, 1196
*R*_int_	0.103
(sin θ/λ)_max_ (Å^−1^)	0.596

Refinement	
*R*[*F* ^2^ > 2σ(*F* ^2^)], *wR*(*F* ^2^), *S*	0.056, 0.094, 0.88
No. of reflections	2726
No. of parameters	199
H-atom treatment	H-atom parameters constrained
Δρ_max_, Δρ_min_ (e Å^−3^)	0.15, −0.20

Computer programs: *X-AREA* and *X-RED* (Stoe & Cie, 2002 ▸), *SHELXT2017* (Sheldrick, 2015*a* ▸), *SHELXL2018* (Sheldrick, 2015*b* ▸), *WinGX* (Farrugia, 2012 ▸), *PLATON* (Spek, 2009 ▸) and *publCIF* (Westrip, 2010 ▸).

## supplementary crystallographic information

### Crystal data

**Table:**

C_17_H_12_Cl_2_N_2_O	*F*(000) = 680
*M**_r_* = 331.19	*D*_x_ = 1.423 Mg m^−^^3^
Monoclinic, *P*2_1_/*c*	Mo *K*α radiation, λ = 0.71073 Å
*a* = 5.8511 (6) Å	Cell parameters from 5578 reflections
*b* = 12.5544 (15) Å	θ = 1.9–30.8°
*c* = 21.069 (2) Å	µ = 0.42 mm^−^^1^
β = 92.666 (8)°	*T* = 296 K
*V* = 1546.0 (3) Å^3^	Stick, colorless
*Z* = 4	0.74 × 0.29 × 0.05 mm

### Data collection

**Table:**

Stoe IPDS 2 diffractometer	2726 independent reflections
Radiation source: sealed X-ray tube, 12 x 0.4 mm long-fine focus	1196 reflections with *I* > 2σ(*I*)
Plane graphite monochromator	*R*_int_ = 0.103
Detector resolution: 6.67 pixels mm^-1^	θ_max_ = 25.0°, θ_min_ = 1.9°
rotation method scans	*h* = −6→6
Absorption correction: integration (X-RED32; Stoe & Cie, 2002)	*k* = −14→14
*T*_min_ = 0.844, *T*_max_ = 0.973	*l* = −25→25
8675 measured reflections	

### Refinement

**Table:**

Refinement on *F*^2^	0 restraints
Least-squares matrix: full	Hydrogen site location: inferred from neighbouring sites
*R*[*F*^2^ > 2σ(*F*^2^)] = 0.056	H-atom parameters constrained
*wR*(*F*^2^) = 0.094	*w* = 1/[σ^2^(*F*_o_^2^) + (0.0186*P*)^2^] where *P* = (*F*_o_^2^ + 2*F*_c_^2^)/3
*S* = 0.88	(Δ/σ)_max_ < 0.001
2726 reflections	Δρ_max_ = 0.15 e Å^−^^3^
199 parameters	Δρ_min_ = −0.20 e Å^−^^3^

### Special details

**Table:**

Geometry. All esds (except the esd in the dihedral angle between two l.s. planes) are estimated using the full covariance matrix. The cell esds are taken into account individually in the estimation of esds in distances, angles and torsion angles; correlations between esds in cell parameters are only used when they are defined by crystal symmetry. An approximate (isotropic) treatment of cell esds is used for estimating esds involving l.s. planes.

### Fractional atomic coordinates and isotropic or equivalent isotropic displacement parameters (Å^2^)

**Table:**

	*x*	*y*	*z*	*U*_iso_*/*U*_eq_	
Cl1	−0.0717 (2)	0.77603 (11)	0.74856 (7)	0.1004 (5)	
Cl2	0.5870 (2)	0.47508 (11)	0.76181 (7)	0.1022 (5)	
O1	−0.0083 (5)	0.4872 (2)	0.58329 (11)	0.0681 (9)	
N1	0.2647 (5)	0.5706 (3)	0.52916 (14)	0.0543 (9)	
H1	0.215035	0.536383	0.495963	0.065*	
N2	0.4383 (5)	0.6397 (3)	0.52057 (14)	0.0497 (8)	
C11	0.5192 (6)	0.6884 (3)	0.57161 (17)	0.0455 (10)	
C9	0.1594 (7)	0.5487 (3)	0.58387 (18)	0.0502 (11)	
C12	0.7076 (6)	0.7653 (3)	0.56287 (18)	0.0486 (10)	
C8	0.2566 (7)	0.6008 (3)	0.63964 (16)	0.0481 (10)	
C10	0.4302 (6)	0.6696 (3)	0.63241 (18)	0.0500 (10)	
H10	0.492982	0.705353	0.667723	0.060*	
C6	0.2580 (7)	0.6252 (4)	0.75974 (16)	0.0496 (10)	
C13	0.8619 (7)	0.7518 (3)	0.51557 (18)	0.0547 (11)	
H13	0.844994	0.694576	0.487703	0.066*	
C1	0.1647 (7)	0.7164 (4)	0.78577 (18)	0.0552 (11)	
C5	0.4515 (7)	0.5861 (3)	0.79161 (19)	0.0587 (11)	
C7	0.1522 (7)	0.5726 (3)	0.70160 (16)	0.0644 (12)	
H7A	−0.008906	0.591153	0.698375	0.077*	
H7B	0.162232	0.496083	0.707307	0.077*	
C17	0.7339 (7)	0.8532 (4)	0.60200 (19)	0.0635 (12)	
H17	0.627740	0.865436	0.632688	0.076*	
C14	1.0395 (7)	0.8220 (4)	0.5094 (2)	0.0652 (13)	
H14	1.141541	0.812120	0.477372	0.078*	
C2	0.2559 (8)	0.7640 (4)	0.8395 (2)	0.0697 (13)	
H2	0.188409	0.824841	0.855430	0.084*	
C15	1.0671 (8)	0.9066 (4)	0.5503 (2)	0.0699 (13)	
H15	1.189954	0.952831	0.546718	0.084*	
C4	0.5475 (8)	0.6332 (5)	0.8459 (2)	0.0791 (15)	
H4	0.678943	0.604863	0.865828	0.095*	
C16	0.9128 (8)	0.9230 (4)	0.5967 (2)	0.0724 (13)	
H16	0.929468	0.980734	0.624227	0.087*	
C3	0.4477 (9)	0.7215 (5)	0.8698 (2)	0.0859 (16)	
H3	0.509354	0.753135	0.906685	0.103*	

### Atomic displacement parameters (Å^2^)

**Table:**

	*U*^11^	*U*^22^	*U*^33^	*U*^12^	*U*^13^	*U*^23^
Cl1	0.0858 (9)	0.0865 (10)	0.1269 (11)	0.0090 (8)	−0.0179 (8)	0.0212 (9)
Cl2	0.1090 (11)	0.0865 (10)	0.1149 (10)	0.0230 (9)	0.0478 (8)	0.0115 (9)
O1	0.083 (2)	0.074 (2)	0.0473 (16)	−0.0306 (18)	−0.0006 (15)	−0.0049 (16)
N1	0.071 (2)	0.053 (2)	0.039 (2)	−0.007 (2)	−0.0005 (18)	−0.0096 (17)
N2	0.055 (2)	0.052 (2)	0.0420 (19)	−0.0023 (18)	0.0037 (16)	−0.0009 (17)
C11	0.049 (2)	0.053 (3)	0.034 (2)	0.003 (2)	−0.0035 (19)	−0.002 (2)
C9	0.061 (3)	0.048 (3)	0.041 (2)	−0.002 (2)	0.002 (2)	0.001 (2)
C12	0.053 (2)	0.050 (3)	0.043 (2)	−0.004 (2)	−0.001 (2)	0.005 (2)
C8	0.058 (2)	0.056 (3)	0.030 (2)	−0.005 (2)	−0.0010 (19)	−0.003 (2)
C10	0.058 (3)	0.055 (3)	0.037 (2)	−0.011 (2)	−0.002 (2)	−0.002 (2)
C6	0.058 (3)	0.061 (3)	0.031 (2)	−0.016 (2)	0.008 (2)	0.000 (2)
C13	0.057 (2)	0.061 (3)	0.046 (2)	−0.002 (2)	0.004 (2)	0.004 (2)
C1	0.063 (3)	0.061 (3)	0.042 (2)	−0.012 (2)	0.004 (2)	0.003 (2)
C5	0.059 (3)	0.067 (3)	0.051 (3)	−0.001 (2)	0.016 (2)	0.008 (2)
C7	0.082 (3)	0.066 (3)	0.046 (2)	−0.022 (3)	0.010 (2)	−0.004 (2)
C17	0.068 (3)	0.070 (3)	0.053 (3)	−0.016 (3)	0.014 (2)	−0.008 (3)
C14	0.055 (3)	0.076 (4)	0.066 (3)	0.007 (3)	0.016 (2)	0.015 (3)
C2	0.091 (3)	0.066 (3)	0.053 (3)	−0.008 (3)	0.014 (3)	−0.011 (3)
C15	0.066 (3)	0.071 (4)	0.073 (3)	−0.014 (3)	0.010 (3)	0.008 (3)
C4	0.069 (3)	0.114 (5)	0.053 (3)	−0.004 (3)	−0.009 (3)	0.011 (3)
C16	0.082 (3)	0.068 (3)	0.068 (3)	−0.019 (3)	0.012 (3)	−0.008 (3)
C3	0.098 (4)	0.117 (5)	0.043 (3)	−0.020 (4)	0.003 (3)	−0.006 (3)

### Geometric parameters (Å, º)

**Table:**

Cl1—C1	1.729 (4)	C13—C14	1.373 (5)
Cl2—C5	1.735 (4)	C13—H13	0.9300
O1—C9	1.248 (4)	C1—C2	1.366 (5)
N1—N2	1.354 (4)	C5—C4	1.383 (5)
N1—C9	1.360 (4)	C7—H7A	0.9700
N1—H1	0.8600	C7—H7B	0.9700
N2—C11	1.307 (4)	C17—C16	1.373 (5)
C11—C10	1.425 (5)	C17—H17	0.9300
C11—C12	1.483 (5)	C14—C15	1.373 (5)
C9—C8	1.438 (5)	C14—H14	0.9300
C12—C17	1.382 (5)	C2—C3	1.373 (6)
C12—C13	1.386 (5)	C2—H2	0.9300
C8—C10	1.348 (5)	C15—C16	1.377 (6)
C8—C7	1.509 (5)	C15—H15	0.9300
C10—H10	0.9300	C4—C3	1.361 (6)
C6—C5	1.380 (5)	C4—H4	0.9300
C6—C1	1.391 (5)	C16—H16	0.9300
C6—C7	1.500 (5)	C3—H3	0.9300
			
N2—N1—C9	128.0 (3)	C6—C5—Cl2	119.2 (3)
N2—N1—H1	116.0	C4—C5—Cl2	117.9 (4)
C9—N1—H1	116.0	C6—C7—C8	115.8 (3)
C11—N2—N1	115.8 (3)	C6—C7—H7A	108.3
N2—C11—C10	121.9 (4)	C8—C7—H7A	108.3
N2—C11—C12	116.4 (4)	C6—C7—H7B	108.3
C10—C11—C12	121.7 (3)	C8—C7—H7B	108.3
O1—C9—N1	120.3 (3)	H7A—C7—H7B	107.4
O1—C9—C8	124.7 (4)	C16—C17—C12	121.7 (4)
N1—C9—C8	115.0 (4)	C16—C17—H17	119.1
C17—C12—C13	117.9 (4)	C12—C17—H17	119.1
C17—C12—C11	120.6 (4)	C13—C14—C15	120.3 (4)
C13—C12—C11	121.5 (4)	C13—C14—H14	119.9
C10—C8—C9	118.1 (4)	C15—C14—H14	119.9
C10—C8—C7	125.8 (3)	C1—C2—C3	119.7 (5)
C9—C8—C7	116.1 (4)	C1—C2—H2	120.2
C8—C10—C11	121.2 (3)	C3—C2—H2	120.2
C8—C10—H10	119.4	C14—C15—C16	120.0 (4)
C11—C10—H10	119.4	C14—C15—H15	120.0
C5—C6—C1	115.4 (3)	C16—C15—H15	120.0
C5—C6—C7	122.6 (4)	C3—C4—C5	119.3 (4)
C1—C6—C7	122.0 (4)	C3—C4—H4	120.3
C14—C13—C12	120.7 (4)	C5—C4—H4	120.3
C14—C13—H13	119.6	C17—C16—C15	119.3 (4)
C12—C13—H13	119.6	C17—C16—H16	120.4
C2—C1—C6	122.8 (4)	C15—C16—H16	120.4
C2—C1—Cl1	117.4 (4)	C4—C3—C2	120.0 (4)
C6—C1—Cl1	119.9 (3)	C4—C3—H3	120.0
C6—C5—C4	122.8 (4)	C2—C3—H3	120.0
			
C9—N1—N2—C11	2.6 (5)	C7—C6—C1—Cl1	−3.3 (5)
N1—N2—C11—C10	0.1 (5)	C1—C6—C5—C4	0.2 (6)
N1—N2—C11—C12	−179.3 (3)	C7—C6—C5—C4	−178.8 (4)
N2—N1—C9—O1	175.7 (3)	C1—C6—C5—Cl2	−178.0 (3)
N2—N1—C9—C8	−4.5 (6)	C7—C6—C5—Cl2	2.9 (5)
N2—C11—C12—C17	149.5 (4)	C5—C6—C7—C8	−83.4 (5)
C10—C11—C12—C17	−29.9 (5)	C1—C6—C7—C8	97.5 (5)
N2—C11—C12—C13	−30.4 (5)	C10—C8—C7—C6	−1.5 (6)
C10—C11—C12—C13	150.1 (4)	C9—C8—C7—C6	178.6 (4)
O1—C9—C8—C10	−176.5 (4)	C13—C12—C17—C16	−3.0 (6)
N1—C9—C8—C10	3.7 (5)	C11—C12—C17—C16	177.1 (4)
O1—C9—C8—C7	3.5 (6)	C12—C13—C14—C15	0.2 (6)
N1—C9—C8—C7	−176.4 (4)	C6—C1—C2—C3	0.1 (6)
C9—C8—C10—C11	−1.5 (6)	Cl1—C1—C2—C3	−178.1 (4)
C7—C8—C10—C11	178.5 (4)	C13—C14—C15—C16	−1.7 (6)
N2—C11—C10—C8	−0.5 (6)	C6—C5—C4—C3	0.6 (7)
C12—C11—C10—C8	179.0 (4)	Cl2—C5—C4—C3	178.8 (4)
C17—C12—C13—C14	2.1 (6)	C12—C17—C16—C15	1.6 (6)
C11—C12—C13—C14	−178.0 (3)	C14—C15—C16—C17	0.9 (7)
C5—C6—C1—C2	−0.6 (6)	C5—C4—C3—C2	−1.1 (7)
C7—C6—C1—C2	178.5 (4)	C1—C2—C3—C4	0.8 (7)
C5—C6—C1—Cl1	177.6 (3)		

### Hydrogen-bond geometry (Å, º)

**Table:**

*D*—H···*A*	*D*—H	H···*A*	*D*···*A*	*D*—H···*A*
N1—H1···O1^i^	0.86	2.04	2.839 (4)	155
C2—H2···O1^ii^	0.93	2.66	3.581 (6)	172

Symmetry codes: (i) −*x*, −*y*+1, −*z*+1; (ii) −*x*, *y*+1/2, −*z*+3/2.
